# Thermal barrier coatings of YSZ developed by plasma sprayed technique and its effective use in orthopedic and dental application

**DOI:** 10.1007/s10856-025-06927-x

**Published:** 2025-11-22

**Authors:** Aishwariya Rajendiran, Vijayalakshmi Uthirapathy

**Affiliations:** Department of Chemistry, School of Advanced Sciences, Vellore Institute of Technology, Vellore, Tamil Nadu India

**Keywords:** YSZ, Plasma spraying, Antibacterial activity, Mechanical studies, Coatings, Orthopedic

## Abstract

The development of durable and biocompatible implant materials remains a critical challenge in the field of biomedical engineering, particularly for dental and orthopedic applications. In this study, yttria-stabilized zirconia (YSZ) coatings with varying molar percentages were deposited on Ti-6Al-4V alloy substrates using the plasma spraying technique. Structural analysis via X-ray diffraction (XRD) confirmed the presence of both transformable and non-transformable phases, with the latter offering enhanced phase stability advantageous for biomedical use. Scanning electron microscopy (SEM) of the cross-sectional morphology revealed that the 5 M% YSZ coating exhibited uniform thickness, low porosity, and absence of cracks, indicating good coating integrity. In vitro hemocompatibility tests with human blood demonstrated a hemolytic ratio below 5%, meeting the threshold for non-hemolytic biomaterials. Antibacterial assays showed notable inhibition against *Escherichia coli*, with moderate activity against *Staphylococcus aureus*. Cytocompatibility was evaluated using MG-63 osteoblast-like cells, where the 5 M% YSZ composite exhibited non-toxic behavior up to 250 µg/mL after 24 h of exposure. Mechanical testing further confirmed the coating’s properties under simulated physiological conditions. These findings suggest that 5 M% YSZ plasma-sprayed coatings present a promising candidate for long-term dental and orthopedic implant applications, owing to their favorable mechanical strength, biocompatibility, and antibacterial properties.

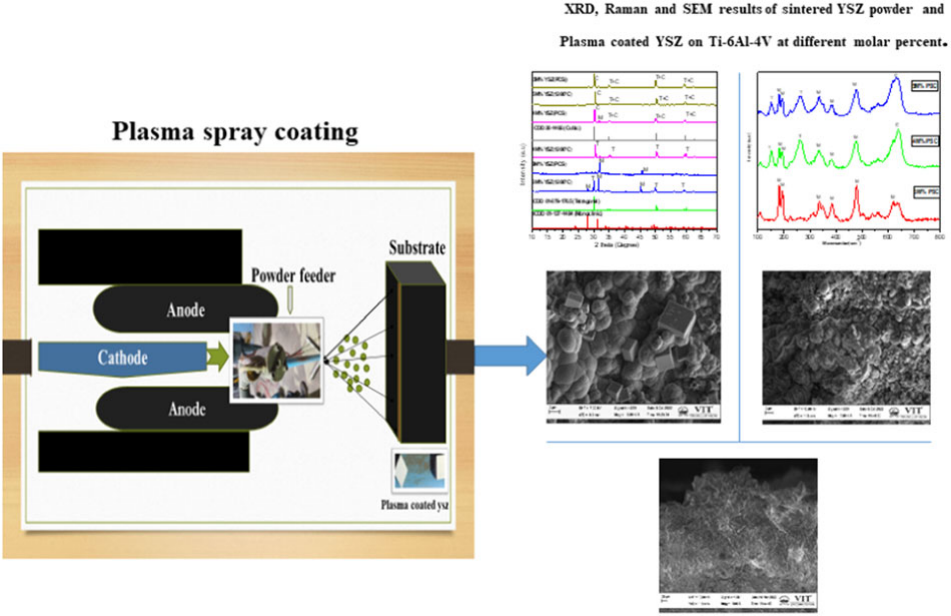

## Highlights

- YSZ was synthesised with varying dopant concentrations and coated onto Ti-6Al-4V alloy using the plasma spray technique.
- The coatings exhibited both tetragonal and cubic phases depending on yttria content.
- Optimized plasma spray parameters produced uniform, crack-free coatings on implant surface.
- YSZ composites demonstrated effective antibacterial activity against *E. coli* and *S. aureus*.
- In vitro biocompatibility studies showed enhanced MG-63 cell proliferation on YSZ composites.

## Introduction

Plasma spraying technique was discovered in early 1980’s, emerged as a preferred method for developing ceramic coatings on metal alloys [1]. It is thermal spray technique that contains high temperature plasma source, typically ranging from 10,000 °C to 15,000 °C. At these temperature, ceramic powder particles melt and are propelled towards the substrate surface. Upon impact, the molten particles rapidly solidify, forming a strong, lamellar- structured coating on the implant surface [2]. In the biomedical field, plasma sprayed bioinert ceramic coatings a promising and widely researched technique. Among these, zirconia and its composites are the most commonly used materials for thermal barrier coatings due to their excellent properties. Zirconia exists in three polymorphic forms at atmospheric pressure: monoclinic, tetragonal, and cubic phases. The monoclinic phase is stable up to approximately 1200 °C, while the tetragonal and cubic phases remain stable up to 2360 °C and 2680 °C, respectively [3]. For biomedical applications, the tetragonal and cubic phases are particularly desirable due to their superior mechanical properties. To stabilize these high-temperature phases at room temperature, two main approaches are commonly employed. (a) Partially Stabilized Zirconia (PSZ) achieved using stabilizing agents such as magnesia or calcia, which have limited solubility at high temperatures. This often results in the precipitation of secondary phases. (b) Tetragonal Zirconia Polycrystals (TZP) formed using stabilizers like yttria or ceria, which possess higher solubility at elevated temperatures, leading to homogeneous solid solutions. These are especially suitable for thermal barrier coatings. Yttria-stabilized zirconia (YSZ) finds extensive applications in load-bearing orthopedic implants, dental restorations, thermal barrier coatings, solid oxide fuel cells (SOFCs), and sensors due to its outstanding physical and mechanical characteristics. Y-TZP (yttria-tetragonal zirconia polycrystal) is known for its exceptional fracture toughness, often reaching values around 1000 MPa, making it one of the most robust ceramic materials. This toughness has made Y-TZP a popular choice in orthopedic implants to mitigate the risk of failure. Despite more than two decades of clinical use, the long-term performance of Y-TZP in orthopedic applications remains a topic of debate among researchers. To address this, modifications and alternative formulations have been explored to reduce implant failure rates. Notably, Y-TZP exhibits resistance to the low-temperature degradation (“aging”) process, making it a reliable option for long-term use in dental applications [4].

High-quality monocrystalline yttria-stabilized zirconia (YSZ) can be synthesized using various techniques, including sol-gel, spray freeze drying, hydrothermal, and co-precipitation methods. However, many of these approaches are limited by drawbacks such as extended processing time, high production costs, and procedural complexity. Among them, the co-precipitation method stands out as a superior choice due to its advantages, including shorter processing time, better control over microstructure, and suitability for large-scale production. It is widely regarded as an efficient and cost-effective technique for synthesizing metal oxide nanoparticles with uniform size and morphology [5, 6].

The processing parameters of implant materials significantly influence their final properties, with sintering being one of the most critical factors. This is especially true for spray-dried powders, where phase transformation is affected by both the yttria content and the sintering temperature. A lower yttria concentration combined with higher sintering temperatures can result in the formation of tetragonal and cubic zirconia phases, which are preferred for biomedical applications due to their superior stability and mechanical properties. Typically, the thickness of plasma-sprayed yttria-stabilized zirconia (YSZ) coatings is less than 500 nm. However, a key drawback of plasma spray techniques is the tendency for crack formation on the coating surface. This issue can be mitigated by optimizing the spraying parameters, including the spraying distance. Despite the advantages of plasma-sprayed coatings, increasing the coating thickness may introduce additional challenges such as it weakens the bonding strength between the ceramic coating and the underlying substrate and prolonged spraying may induce residual stress within the coating, compromising its integrity [7, 8]. Addressing these limitations while developing alternative coating materials remains a complex task. Various studies have investigated the antibacterial potential of YSZ coatings. For instance, Uchida et al. reported that zirconia coatings fabricated via hard-coating methods, such as cathodic arc deposition and micro-arc oxidation, exhibited bioactive properties. Their findings challenge the traditional classification of zirconia as completely bioinert; instead, certain zirconia-based materials have shown bioactivity, particularly through the formation of apatite via surface Zr–OH groups.

These Zr–OH groups, when chemically treated with acids such as phosphoric, sulfuric, or hydrochloric acid or with alkaline solutions like sodium hydroxide on zirconia/alumina composites, were found to induce apatite formation in simulated body fluid (SBF). Notably, YSZ composites with yttria content greater than 5 mol% treated in NaOH showed superior apatite-forming ability compared to other compositions [9].

Moreover, recent studies have investigated the anticancer, antioxidant, and antibacterial properties of zirconia (ZrO_2_) nanoparticles for potential biomedical applications. Antibacterial assays conducted against *Staphylococcus aureus* and *Escherichia coli* demonstrated that doped ZrO_2_ nanoparticles produced larger inhibition zones compared to undoped samples, indicating enhanced antibacterial efficacy. For instance, the study reported that Fe_3_O_4_-doped ZrO_2_ nanoparticles exhibited superior antibacterial activity against *S. aureus*, *E. coli*, and *Bacillus subtilis* [10, 11]. Similarly, Jangra et al. found that 5 M% yttria-stabilized zirconia (YSZ) nanoparticles showed pronounced antibacterial effects, particularly against *E. coli* [11]. Additionally, Thirumagal et al. observed that *S. aureus* produced an inhibition zone of 8 mm at lower concentrations of the material, while *Streptococcus mutans* exhibited an 11 mm inhibition zone at higher concentrations [12].

This study presents the fabrication of composite ceramic coatings on Ti-6Al-4V alloy using 3 M%, 4 M%, and 5 M% yttria-stabilized zirconia (YSZ) composites. Optimized plasma spray parameters were established to achieve crack-free coatings with appropriate thickness. To the best of our knowledge, limited studies have explored the antibacterial behavior of YSZ composites against *Escherichia coli* and *Staphylococcus aureus*. Therefore, for the first time, we report the synthesis of YSZ powders with varying yttria concentrations (3 M%, 4 M%, and 5 M%) using the co-precipitation method, followed by plasma spray coating on Ti-6Al-4V substrates. The synthesized powders and coatings were characterized using Fourier-transform infrared spectroscopy (FTIR), X-ray diffraction (XRD), and scanning electron microscopy with energy-dispersive X-ray analysis (SEM-EDAX). Furthermore, we conducted in vitro assessments of hemocompatibility, antibacterial activity, and biocompatibility using MG-63 osteoblast-like cell lines. Mechanical testing was also performed to evaluate the influence of yttria addition on the structural and functional performance of zirconia-based coatings for biomedical applications.

## Materials and methods

### Synthesis of Zirconia

Zirconium oxychloride octahydrate (ZrOCl_2_·8H_2_O) was dissolved in 100 ml of distilled water. Separately, 3 M%, 4 M%, and 5 M% of yttrium oxide (Y_2_O_3_) were each dissolved in 100 ml of distilled water. 1 mol% solution of ZrOCl_2_·8H_2_O was used as the zirconium precursor, and the yttria solution was added dropwise as the stabilizing agent. After 15–20 min of stirring, 2 mol% sodium hydroxide (NaOH) solution was added dropwise until the pH of the mixture reached 9. The solution was then stirred continuously for 2 h and allowed to undergo aging for 24 h at room temperature. Following the aging process, the precipitate was washed several times with distilled water and acetone to remove any residual acid contaminants. The resulting material was then filtered and dried in an oven at 60–70 °C for 24 h.

### Surface treatment of metal sample for plasma spraying

Ti-6Al-4V alloy was used as the substrate material. The alloy was cut into specimens measuring 1 × 1 cm with a thickness of 5 mm. Prior to coating, the substrates were polished using a series of silicon carbide (SiC) abrasive papers of varying grit sizes, followed by final polishing with a diamond suspension and alumina paste to achieve a smooth surface finish. The polished substrates were then rinsed thoroughly with distilled water and acetone to remove any surface contaminants. Subsequently, the samples were ultrasonicated in acetone for 20 min to ensure complete cleaning. After sonication, the substrates were dried in a hot air oven at 60 °C for a few minutes and then prepared for the plasma spray coating process.

**Fig. 1:**
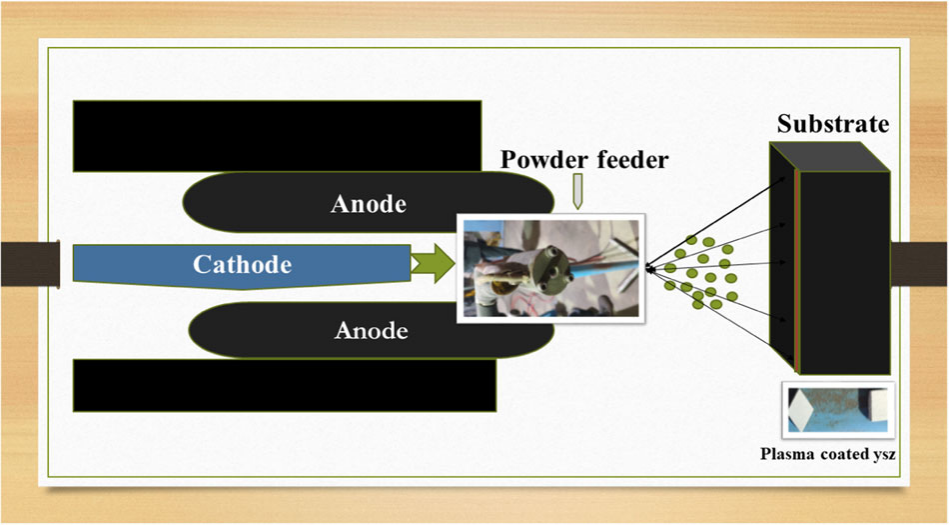
Schematic diagram of Plasma spray coating

**A schematic representation of the plasma spray setup is shown in** Fig. 1.

The optimized parameters for the plasma spray process included the use of argon and hydrogen gases as the primary and auxiliary plasma-forming gases, respectively. The plasma arc was maintained at a current of 620 A and a voltage of 67 V. The powder feed rate was set at approximately 20 g/min. The spraying was carried out at an air pressure of 5.2 bar, with a spray distance of 1.5 cm between the nozzle and the substrate surface. The detailed plasma spraying parameters are summarized in Table 1.

**Table 1:** Optimized parameters for plasma spray process

Amp(A)/Volts(V)	580	600	620
Argon flow(l/min)	90	80–90	50
Hydrogen flow(l/min)	16	20	14
Carrier gas flow(l/min)	15	15	14
Powder feed rate(gm/min)	36	37	20
Air pressure (bar)	5.85	5.8	5.2
Spray distance(mm)	140	130	120
Optimized parameters	Coating is not uniform, voids are formed.	Coating is not uniform, crack was formed.	Optimized coating parameter.

## Characterization techniques

### Microstructure and mechanical analysis

X-ray diffraction (XRD) analysis was carried out using a Bruker D8 Advance diffractometer (Germany) to investigate the phase transformations of the synthesized materials. The measurements were performed using Cu Kα radiation (λ = 1.5406 Å) at an operating voltage of 30 kV and a current of 40 mA, over a 2θ range of 0° to 90°. Fourier-transform infrared (FTIR) spectroscopy was conducted using an IR Affinity-1 spectrophotometer (Shimadzu, Japan) to identify the functional groups present in the 3 M%, 4 M%, and 5 M% YSZ powders. The spectra were recorded in the range of 4000–400 cm^−1^. Scanning electron microscopy (SEM) was employed to examine the surface morphology of the synthesized and coated samples, while energy-dispersive X-ray spectroscopy (EDS) was used to determine the elemental composition. Cross-sectional analyses of the coatings were performed at an accelerating voltage of 30 kV using SEM–EDS. The micro hardness of the YSZ coatings deposited on Ti-6Al-4V substrates was evaluated using a Vickers micro hardness tester. A load ranging from 0.1 kgf to 0.5 kgf was applied to the coating surface with a dwell time of 15 seconds. The Vickers hardness (Hv) was calculated using the following formula:

$${{\rm{H}}}_{{\rm{v}}}=1.844{\rm{P}}/{{\rm{d}}}^{2}({{\rm{kg}}/{\rm{mm}}}^{2})$$

Where, d= average length of indentation in mm.

1.854 = Geometrical constant of diamond.

### Biological properties

#### In vitro hemocompatibility

In vitro hemocompatibility was assessed using a 5 mL blood sample voluntarily donated by a healthy 26-year-old male from the research group (Health Center, VIT Vellore, India: Ref. No. VIT/IECH/XIII/2023/17) [11–14]. The collected blood was used to evaluate the hemolytic activity of pure zirconia and yttria-stabilized zirconia (YSZ) powders. The blood sample was first centrifuged at 10,000 rpm for 2–3 min to separate the plasma from red blood cells (RBCs). The isolated RBCs were then washed with freshly prepared phosphate-buffered saline (PBS) and centrifuged at 10,000 rpm for 4 min at 5 °C. This washing and centrifugation cycle was repeated 3–4 times using 5 mL of PBS each time to thoroughly purify the RBCs. After washing, the RBCs were suspended in 15 mL of PBS and stored under refrigeration until use. For the hemolysis test, 0.2 mL of the RBC suspension was mixed with 0.8 mL of PBS containing either pure zirconia or YSZ powders at different concentrations. The mixtures were stirred and incubated at 37 °C for 1 h. Following the incubation period, the samples were centrifuged, and the supernatant was collected for absorbance measurements. Hemoglobin release was quantified using a Bio-Rad ELISA reader at 570/655 nm. To establish controls, 0.2 mL of RBC suspension was mixed with deionized water (positive control) and PBS (negative control), respectively. The examinations were carried out three times to ensure reliable results.

The percentage of hemolysis (hemolytic ratio) was calculated using the following formula:

$${\rm{Hemolytic}} \% =\frac{({\rm{Abs\; of\; sample}}-{\rm{Abs\; of\; negative}})}{({\rm{Abs\; of\; positive}}-{\rm{Abs\; of\; negative}})}$$

#### Antibacterial activity

The antibacterial properties of pure zirconia and yttria-stabilized zirconia (YSZ) composites used for coatings with 3 M%, 4 M%, and 5 M% yttria content were evaluated using the agar disc diffusion method. The bacterial strains selected for this study were *Staphylococcus aureus* (*S. aureus*) and *Escherichia coli* (*E. coli*), which are commonly associated with implant-related infections and post-surgical complications [15]. Nutrient agar plates were prepared and inoculated under aseptic conditions. A 100 µl aliquot of the overnight bacterial cultures was uniformly spread on the agar surface using a sterile glass L-rod. Sterile 10 mm diameter paper discs (Whatman filter paper) were soaked in a 5 mg/ml solution of each test sample (pure zirconia, 3 M%, 4 M%, and 5 M% YSZ), dissolved in 10% DMSO. The discs were then carefully placed onto the surface of the inoculated plates. The plates were incubated at 37 °C for 24 h to allow for bacterial growth. After incubation, the zone of inhibition around each disc was measured using a ruler to assess the antibacterial effectiveness of each material. The minimum inhibitory concentration (MIC) was determined as the lowest concentration that inhibited visible bacterial growth. All experiments were conducted in triplicate to ensure reproducibility. Chloramphenicol was used as the positive control, and distilled water served as the negative control (dissolving agent for the extracts) [13].

#### In vitro biocompatibility analysis

The cytotoxicity of the YSZ-composites used for coatings was evaluated using the MG-63 human osteosarcoma cell line. The materials and reagents used for the assay included Modified Eagle’s Medium (MEM), fetal bovine serum (FBS), phosphate-buffered saline (PBS), cell culture grade dimethyl sulfoxide (DMSO), doxorubicin (as the positive control), and the MTT reagent: 3-(4,5-dimethyl-2-thiazolyl)-2,5-diphenyl-2H-tetrazolium bromide. The biomaterial extract was prepared by incubating the YSZ samples (3 M% and 5 M%) in MEM supplemented with 10% FBS and doxorubicin. The extract solutions were diluted to various concentrations using freshly prepared MEM for the cytotoxicity tests.

MG-63 cells were cultured in MEM supplemented with 10% FBS, 1× antimycotic solution, and doxorubicin under standard culture conditions at 37 °C in a humidified atmosphere containing 5% CO_2_. The cells were then seeded into 96-well plates at a density of 1 × 10^4^ cells per well. After cell attachment, the media were replaced with sample extracts of varying concentrations along with 1× PBS-washed cells. Doxorubicin (at its IC_50_ concentration) in serum-free media was used as a positive control. The plates were incubated for 24 h. Following incubation, 0.5 mg/mL of MTT solution was added to each well, and the plates were further incubated for 4 h at 37 °C. After incubation, the MTT-containing media were carefully removed, and 200 µl of PBS was used to rinse the wells. The resulting formazan crystals were solubilized using 100 µl of DMSO, and the contents were mixed thoroughly. The absorbance of the resulting purple color, indicative of viable cells, was measured at 570 nm using a microplate reader. All experiments were conducted in triplicate to ensure reproducibility. Cell viability was calculated based on the optical density (OD) values using the following formula [14]:

$${\rm{Cell\; viability}} \% =\frac{{\rm{Intensity\; of\; the\; sample}}}{{\rm{Intensity\; of\; the\; control}}}{\rm{X}}100$$

#### Statistical analysis

The Antibacterial activity and in vitro biocompatibility studies were conducted to evaluate the biological properties of 3 to 5 M% yttria-stabilized zirconia (YSZ). All experiments were performed in triplicate to ensure data reliability, and the results are presented as mean ± standard deviation (SD). Statistical analysis was carried out using GraphPad Prism 6.0 software. Two-way ANOVA followed by Tukey’s multiple comparison test was applied to assess statistical significance, with *P* < 0.05 considered statistically significant.

## Results and discussion

### FT-IR spectroscopy

**Fig. 2:**
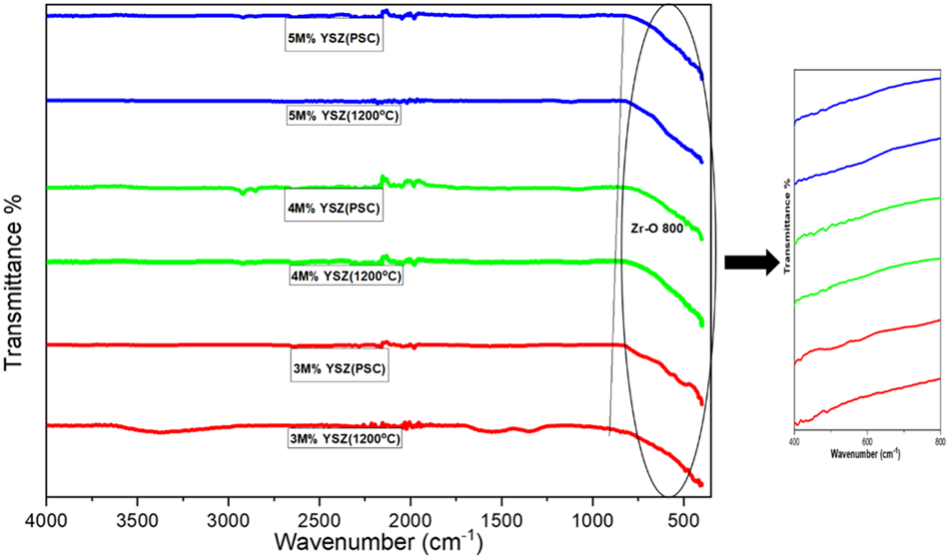
FT-IR analysis for 3 M%, 4 M% and 5 M% sintered powder and plasma coated YSZ

The functional groups present in the synthesized yttria-stabilized zirconia (YSZ) powders and their corresponding plasma-sprayed coatings were analysed using Fourier-transform infrared (FTIR) spectroscopy. Figure 2 displays the FTIR spectra of both uncoated and coated YSZ samples with varying molar concentrations of yttria. For the 3 M% YSZ powder, a broad absorption band at 3373 cm^−1^ was observed, corresponding to the O–H stretching vibration, which indicates the presence of adsorbed moisture on the surface. This band was absent in the coated sample, indicating that plasma spraying at elevated temperatures effectively removed surface-bound water. A peak at 1661 cm^−1^ was assigned to the C–O bending vibrations, also attributed to residual water molecules [4]. The sharp band at 2300 cm^−1^ is associated with C = O stretching, likely arising from atmospheric CO_2_ adsorption or moisture-derived carbonate groups, a common observation in thermally treated zirconia ceramics [15]. The broad absorption band between 1227–1700 cm^−1^ is indicative of carbonate or ionic carbonate species, which persist post-sintering and coating, possibly due to surface reactions during thermal exposure [16]. While C–O and O–H bands were still observed in small intensities post-coating, their reduced peak areas suggest partial decomposition or volatilization during the plasma spraying process. Additionally, it was noted that the intensity of some absorption bands increased slightly with rising Y_2_O_3_ concentration; however, these differences were not significant enough to be clearly distinguished in the spectra. The vibrational modes characteristic of zirconia was clearly observed in the 400–1200 cm^−1^ region, particularly the metal–oxygen (Zr–O and Y–O) stretching modes. These bands were most prominent in the 500–800 cm^−1^ region. A strong absorption peak around 800 cm^−1^ confirms the successful incorporation of yttrium into the zirconia matrix and the formation of YSZ in both powder and coated samples. These results are consistent with previous findings reported by Joshi et al., who documented metal–oxygen bond vibrations in the 500–800 cm^−1^ range for plasma-sprayed zirconia coatings [17], and Padovini et al., who observed carbonate-related bands in thermally treated zirconia composites [18].

### XRD patterns of YSZ powder samples and its plasma sprayed coatings

**Fig. 3:**
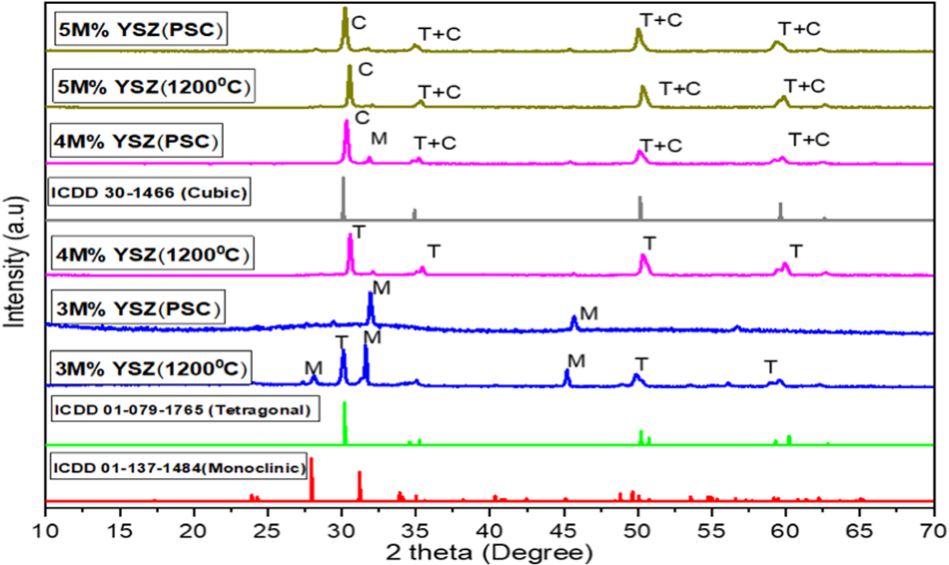
XRD patterns of sintered YSZ powder and Plasma coated YSZ on Ti-6Al-4V at different molar percent

The XRD patterns of the synthesized yttria-stabilized zirconia (YSZ) powders and their corresponding plasma-sprayed coatings revealed a mixed-phase composition, with the presence of both tetragonal (t-ZrO_2_) and cubic (c-ZrO_2_) phases when sintered at 1200 °C. These findings are consistent with previous studies reporting that sintering temperatures beyond 1200 °C can lead to the formation of the monoclinic (m-ZrO_2_) phase, which is typically undesired in biomedical applications due to its lower mechanical stability and transformation-induced volume change [19]. The observed diffraction peaks correspond well with standard reference data from the ICDD database: 01-137-1484 (monoclinic), 01-079-1765 (tetragonal), and 30-1466 (cubic). Figure 3 presents the XRD spectra of both sintered powders and plasma-sprayed coatings at 3 M%, 4 M%, and 5 M% Y_2_O_3_ doping concentrations. The phase composition varied with yttria content, where higher concentrations promoted the stabilization of high-temperature phases (tetragonal and cubic). 3 M% YSZ powder exhibited a combination of monoclinic and tetragonal phases, indicated by strong peaks at 2θ = 28.3°, 31.2°, and 41.1° (monoclinic), and minor peaks at 29.9°, 49.7°, and 59.9° (tetragonal). In the 3 M% YSZ coating, a dominant monoclinic phase was detected. This suggests that thermal shock and cooling rates during plasma spraying caused a partial reverse transformation from tetragonal to monoclinic, as also noted by Guo et al. [20–22]. 4 M% YSZ powder primarily displayed the tetragonal phase, whereas the coated sample revealed a mixture of tetragonal and cubic phases, with minor monoclinic traces highlighting the influence of dopant content and plasma energy on phase transformation. At 5 M% Y_2_O_3_, both powder and coating showed pure tetragonal and cubic phases, without notable monoclinic presence. This signifies that sufficient Y^3+^ ions effectively stabilize the zirconia lattice, suppressing undesirable phase transitions during the coating process. These results reinforce earlier conclusions by Garvie et al. and Piconi & Maccauro that 3–5 M% Y_2_O_3_ stabilizes tetragonal/cubic phases at room temperature, but our study improves upon this by: Using plasma spraying with pre-sintered powders to minimize phase degradation, demonstrating coating integrity and phase retention at 5 M%, confirming minimal monoclinic back-transformation Tables 2.

**Table 2:** represents the comparison of phases with respect to various molar percentage of Y_2_O_3_ for the powder and coated samples

Sample code	Concentration of precursor (M %)	Concentration of dopant (M %)	Phase transformations	
			Sintered powder	Plasma coating
3 M% YSZ	1	0.03	M + T	M + M
4 M% YSZ	1	0.04	T	M + T + C
5 M% YSZ	1	0.05	T + C	T + C

Additionally, the long-term durability of YSZ coatings is strongly connected to this phase stability. The suppression of monoclinic transformation under thermal or mechanical stress ensures that the coating maintains high fracture toughness, wear resistance, and chemical inertness over extended periods. In biomedical applications, particularly under dynamic physiological conditions, monoclinic reversion can lead to micro cracking and particle release, which compromise implant integrity and biocompatibility. The dominance of stable tetragonal and cubic phases in the 5 M% YSZ coating suggests enhanced resistance to hydrothermal aging, mechanical fatigue, and phase transformation, thus supporting its suitability for long-term implantation. These characteristics align with durability studies by Trice et al. and Langjahr et al., who emphasized the role of optimized Y_2_O_3_ doping and controlled microstructures in maintaining coating performance under cyclic or prolonged service conditions. Therefore, this work not only confirms the phase evolution behavior across Y_2_O_3_ doping levels but also highlights the significance of 5 M% YSZ in achieving a durable, phase-stable, and structurally strong coating critical for long-term biomedical and structural applications [23, 24].

### Raman spectroscopy

**Fig. 4:**
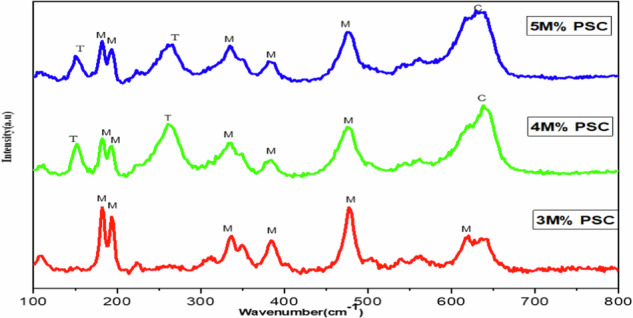
Raman spectra of 3 M%, 4 M% and 5 M% of plasma sprayed coating of YSZ on Ti-6Al-4V

Raman spectroscopy was employed to complement the X-ray diffraction (XRD) results and provide deeper insight into the phase composition of the Yttria-Stabilized Zirconia (YSZ) coatings. Figure 4 displays the Raman spectra of YSZ coatings with 3 M%, 4 M%, and 5 M% Y_2_O_3_, deposited on Ti-6Al-4V substrates. The spectra revealed vibrational modes characteristic of the monoclinic, tetragonal, and cubic phases of zirconia. The monoclinic phase was identified by its signature peaks at 182, 191, 336, 384, 475, and 620 cm^−1^. The tetragonal phase (t-YSZ) was represented by bands at 151 and 262 cm^−1^, while a prominent peak at 637 cm^−1^ was attributed to the cubic phase (c-YSZ) [25, 26]. The 3 M% YSZ coating predominantly exhibited monoclinic features, indicating incomplete stabilization. With increasing Y_2_O_3_ content to 4 M% and 5 M%, the intensities of the tetragonal and cubic peaks progressively increased, suggesting a dopant-induced phase transformation. Although some of the peaks remained largely unchanged, minor spectral variations below 200 cm^−1^ were observed, likely due to oxygen vacancies introduced during yttria doping, which perturb the vibrational modes of the crystal lattice. All coatings showed a mixed-phase composition, but a clear trend emerged, increasing yttria content led to a higher fraction of tetragonal and cubic phases. Notably, the 5 M% YSZ coating showed dominant tetragonal and cubic features, consistent with XRD analysis. To estimate the relative cubic phase content, the Clarke and Adar [27]

$${vc}=\frac{0.97[{\rm{Ic}}(637)]}{0.97[{\rm{Ic}}(637)]+{{\rm{I}}}_{{\rm{t}}}(475)]}$$

Where,

Vc is the volume fraction of the cubic phase, Ic(637) is the intensity of the cubic peak at 637 cm^−1^, I_t_(475) is the intensity of the tetragonal peak at 475 cm^−1^. Based on this analysis, the cubic phase ratios were found to be: 54.16% for 3 M% YSZ, 61.87% for 4 M% YSZ, 67.16% for 5 M% YSZ. These results confirm that higher yttria concentrations promote cubic phase formation in the zirconia lattice, with Raman and XRD analyses showing excellent correlation in identifying and quantifying the phase evolution.

### Morphological and microstructural analysis

**Fig. 5:**
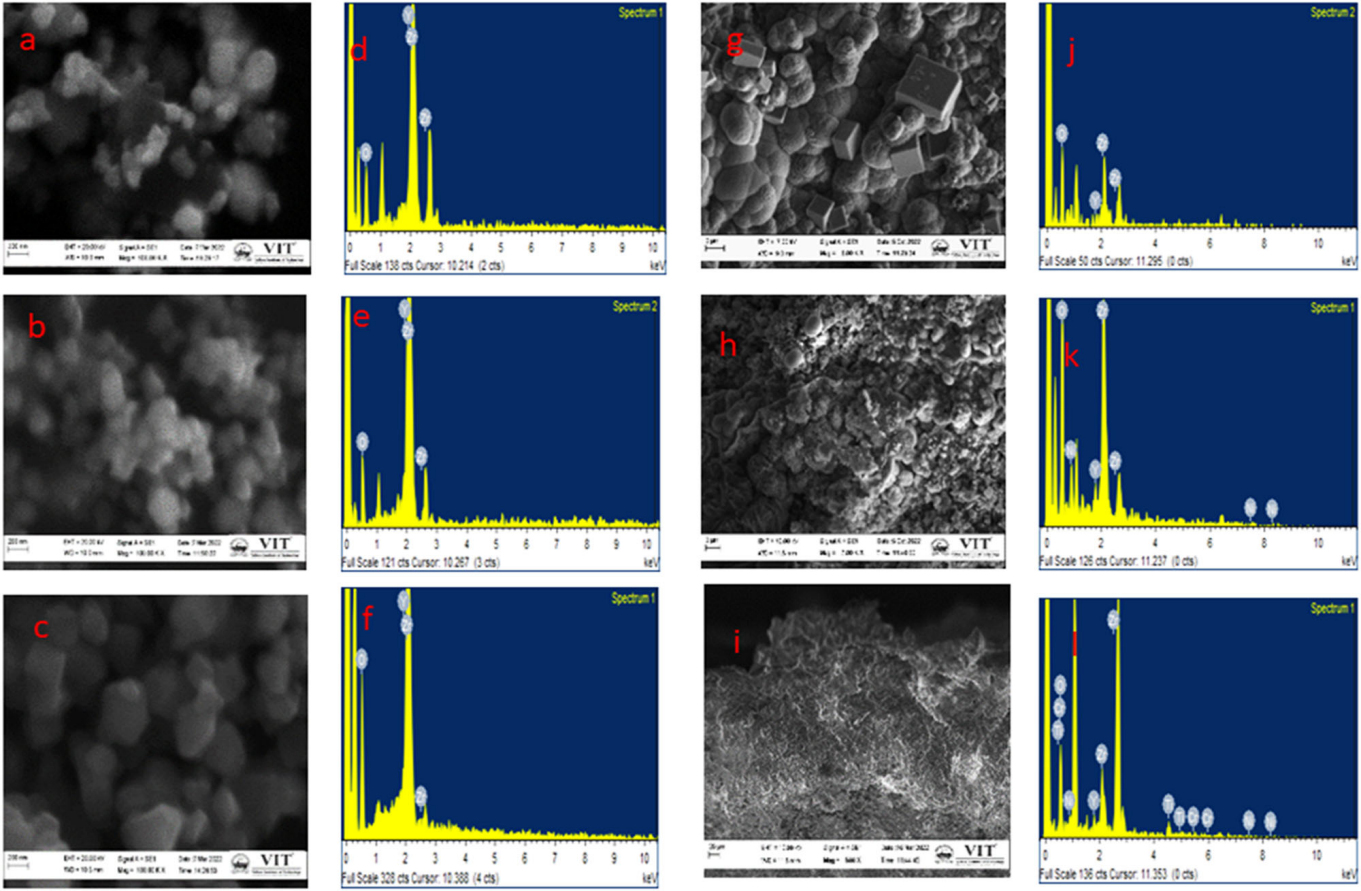
SEM morphology **a** 3 M% YSZ **b** 4 M% YSZ **c** 5 M% YSZ powders, **g** 3 M% YSZ coating, **h** 4 M% YSZ coating and **i**) 5 M% YSZ coating, EDAX of (**d**) 3 M% YSZ **e** 4 M% YSZ **f** 5 M% YSZ powders **j** 3 M% YSZ coating **k** 4 M% YSZ coating and **l** 5 M% YSZ coating

Figure 5 presents the SEM micrographs of YSZ powders with varying Y_2_O_3_ concentrations and their corresponding plasma-sprayed coatings. As observed, increasing yttria concentration led to more noticeable particle agglomeration and reduction in average particle size. Specifically, the 5 M% YSZ powder displayed a more uniform morphology, although mild agglomeration persisted an effect previously attributed to enhanced lattice stabilization and finer grain size due to higher Y_2_O_3_ doping [28, 29]. In the case of plasma-sprayed coatings, the 3 M% YSZ sample exhibited a bimodal surface structure composed of both fully and partially melted particles characteristic of conventional plasma spray processes. The distinct black and white contrast in the SEM images reflects dopant (yttria) inhomogeneity within the zirconia matrix, corroborating earlier findings of differential melting behavior during rapid solidification [30]. Although the 3 M% YSZ coating appeared relatively dense with minimal surface porosity, longitudinal micro cracks were observed (Fig. 5g, h), likely due to unmelted particles and low-temperature sintering effects, which induce residual stress during rapid thermal cycling [31]. In contrast, the 4 M% YSZ coating showed more pronounced porosity and voids, indicating inadequate melting and suboptimal cohesion among splats. Despite a marginal improvement in particle distribution and layer thickness compared to the 3 M% sample, the overall coating morphology remained less homogeneous. The 5 M% YSZ coating emerged as the most optimized among the studied compositions. It displayed uniform particle distribution, a dense and crack-free microstructure, and no visible grooves or delamination (Fig. 5i). The enhanced coating quality at this dopant level can be attributed to improved thermal conductivity and melting behavior, which promote complete fusion and densification during deposition [32, 33]. Additionally, the pre-sintering of YSZ powders at 1200 °C likely contributed to crack suppression and improved lamellar bonding, aligning with the multi-stage sintering mechanism proposed in earlier studies [34, 35]. In the initial sintering stage (within 20 h), point contacts at lamellae and micro-fissures serve as diffusion channels, facilitating the gradual healing of cracks and pores. As sintering progresses, mass transport slows due to decreased diffusion sites, highlighting the critical role of initial microstructural design. Based on these SEM analyses and morphological features, the 5 M% YSZ composition is deemed most suitable for high-performance biomedical coatings, particularly in dental and orthopedic implant applications, where surface integrity, mechanical durability, and microstructural stability are crucial Tables 3.

**Table 3:** Elemental composition of powder and coated YSZ:

Elements (%)	3 M% YSZ	4 M% YSZ	5 M% YSZ	3 M% plasma coated YSZ	4 M% plasma coated YSZ	5 M% plasma coated YSZ
O K	38.47	34.78	33.68	33.65	32.64	33.64
Y L	2.97	4.03	5.04	1.94	3.97	4.31
Zr L	58.56	61.20	61.20	21.96	20.12	22.14
Cr L	–	–	–	19.39	19.03	24.03
Ni L				23.06	24.24	15.88
Total	100.00	100.00	100.00	100.00	100.00	100.00

**Fig. 6:**
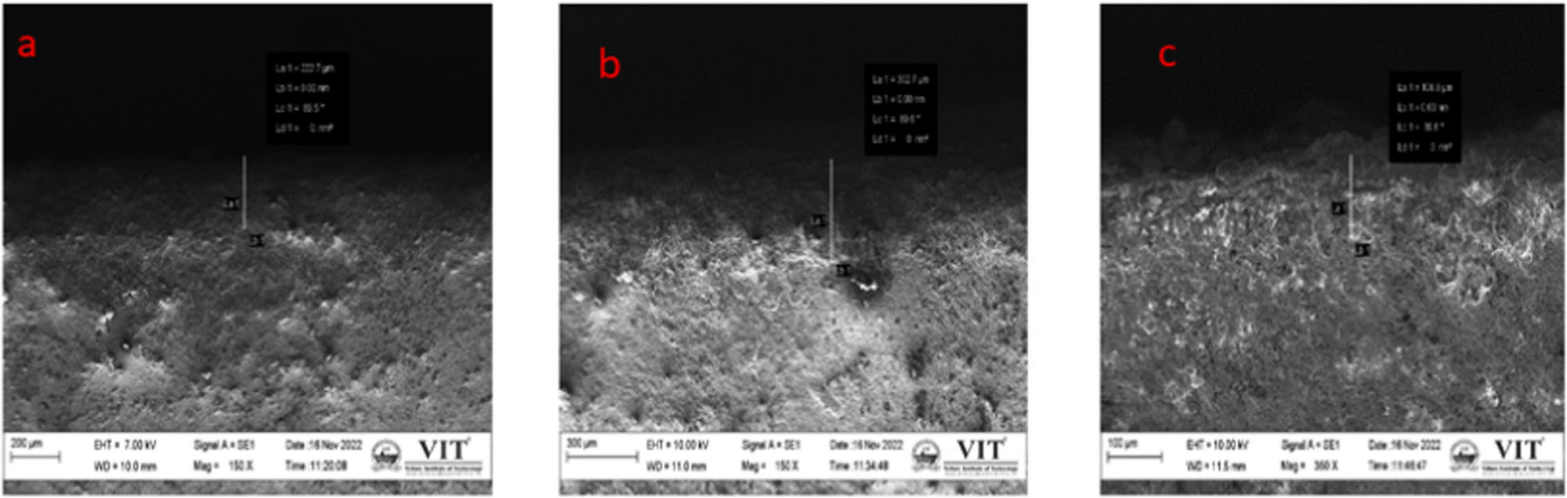
Cross sectional of (**a**) 3 M% ysz **b** 4 M% ysz **c** 5 M% ysz cross sectional coatings **d** EDAX of (i) 3 M%, (ii) 4 M%, (iii) 5 M% YSZ coating

The thickness of the plasma-sprayed YSZ coatings was evaluated from SEM cross-sectional images. The measured thickness values for 3 M%, 4 M%, and 5 M% YSZ coatings were 227.7 µm, 302.7 µm, and 108.8 µm, respectively. From the cross-sectional SEM micrographs, two distinct layers were clearly identified: the Ti-6Al-4V alloy substrate and the ceramic coating layer. The coating thickness was found to be highly influenced by the spraying distance, a key parameter in the plasma spraying process. Among the tested distances, a 120 mm spraying distance was optimized to achieve a uniform and adherent coating layer. The 4 M% YSZ coating exhibited the maximum thickness of 302.7 µm, which corresponded to optimal process conditions. However, the 5 M% YSZ coating, despite having a lower thickness of 108.8 µm, demonstrated superior uniformity and surface quality. This may be attributed to the smaller particle size and improved flowability of the highly doped YSZ powder, which enabled better melting and deposition during spraying. The thinner but more uniform 5 M% coating also exhibited excellent adhesion, free of delamination, pores, or cracks, suggesting it is structurally more stable. This supports the conclusion that 5 M% YSZ, despite yielding a thinner layer, produces the most mechanically and morphologically strongest coating, suitable for long-term biomedical applications Figs. 6.

### Surface roughness

**Fig. 7:**
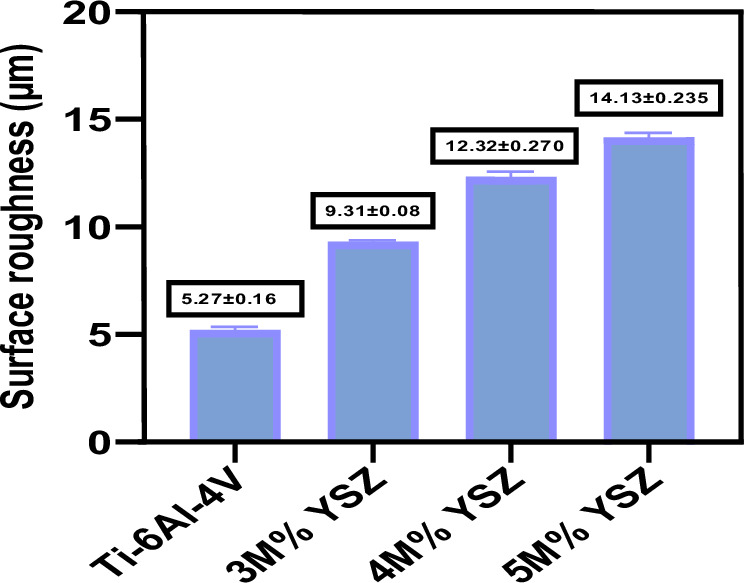
Surface roughness measurement of Ti-6Al-4V, 3 M%, 4 M% and 5 M% ysz coatings

The surface roughness of the uncoated Ti-6Al-4V and YSZ coating was analyzed and was mentioned in Table 4. The polished Ti-6Al-4V attains a smooth surface and showed the value of 5.27 ± 0.165 µm. The different concentration of YSZ coatings exhibited different surface roughness of 9.31 ± 0.087, 12.3 ± 0.270, 14 ± 0.235 µm respectively Figs 7. According to Deligianni et al., increasing roughness promotes cell attachment and proliferation thereby, strengthening the bone implant’s adherence. [36]

**Table 4:** Surface roughness and thickness of the coating:

Coating code	Surface roughness (µm)	Thickness of the coating (µm)
Ti-6Al-4V	5.27 ± 0.165	–
3M% YSZ	9.31 ± 0.087	227.7
4M% YSZ	12.32 ± 0.270	302.7
5M% YSZ	14.13 ± 0.235	108.8

### Wettability

**Fig. 8:**
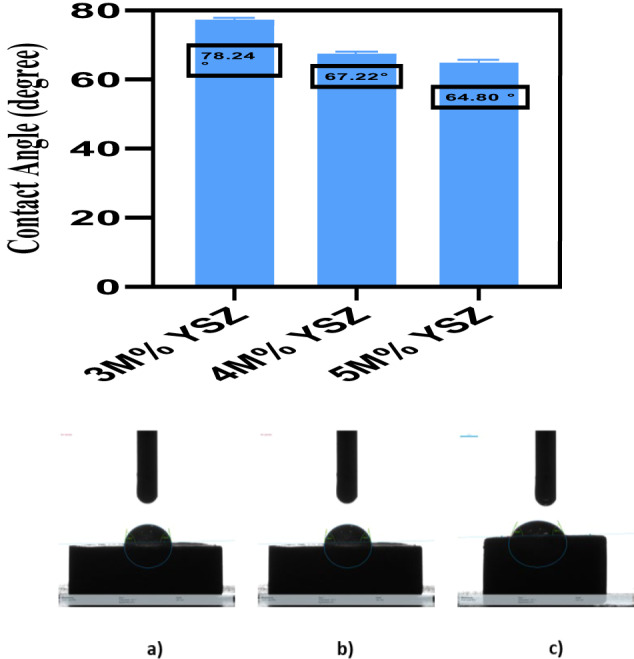
Contact angle measurement of (**a**) 3 M%, (**b)** 4 M%, and (**c)** 5 M% YSZ coatings

The contact angle measurements were carried out on plasma-sprayed YSZ coatings to evaluate surface wettability at different Y_2_O_3_ concentrations using the sessile drop method (Fig. 8). The measured contact angles for the 3 M%, 4 M%, and 5 M% YSZ coatings were 78.24°, 67.22°, and 64.8°, respectively. According to Yu et al. [37], the presence of hydroxyl groups on the oxide surface hydrogen bonding with water molecules, thereby enhancing the hydrophilic nature of the surface. All three YSZ coatings exhibited contact angles below 90°, indicating hydrophilic behavior. Notably, the 5 M% YSZ coating demonstrated the lowest contact angle (64.8°), signifying the best wettability among the three. This enhanced wettability can be attributed to the increased stability of the non-transformable tetragonal phase at higher Y_2_O_3_ concentrations, which results in fewer phase transformations and reduced surface micro cracking. SEM analysis confirmed that the 5 M% YSZ coatings were relatively uniform but exhibited minor surface voids. These voids may facilitate the penetration of water droplets, further contributing to improved wettability. In contrast, the 3 M% and 4 M% YSZ coatings displayed slightly higher contact angles (78.24° and 67.22°, respectively), which may be linked to the presence of both micro cracks and voids observed in the SEM micrographs. The increase in surface roughness, especially in the 5 M% coatings, is also known to enhance hydrophilicity by reducing the contact angle, which is beneficial for cell attachment and proliferation key factors in orthopedic and dental implant applications [38]. Thus, the 5 M% YSZ coating not only exhibits optimal wettability but also maintains surface integrity, making it a promising candidate for bioactive implant surfaces.

### Vickers micro-hardness

**Fig. 9:**
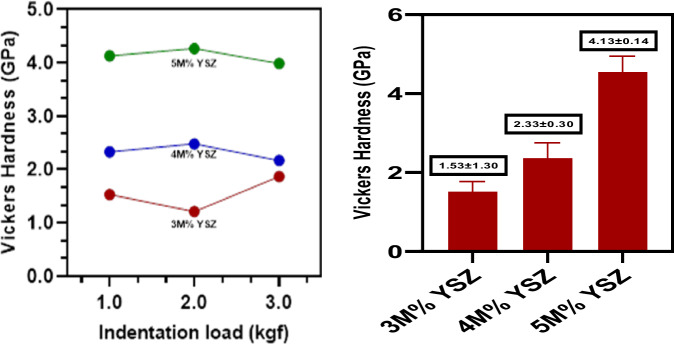
Vickers micro hardness test of 3 M%, 4 M% and 5 M% YSZ coatings

**Fig. 10:**
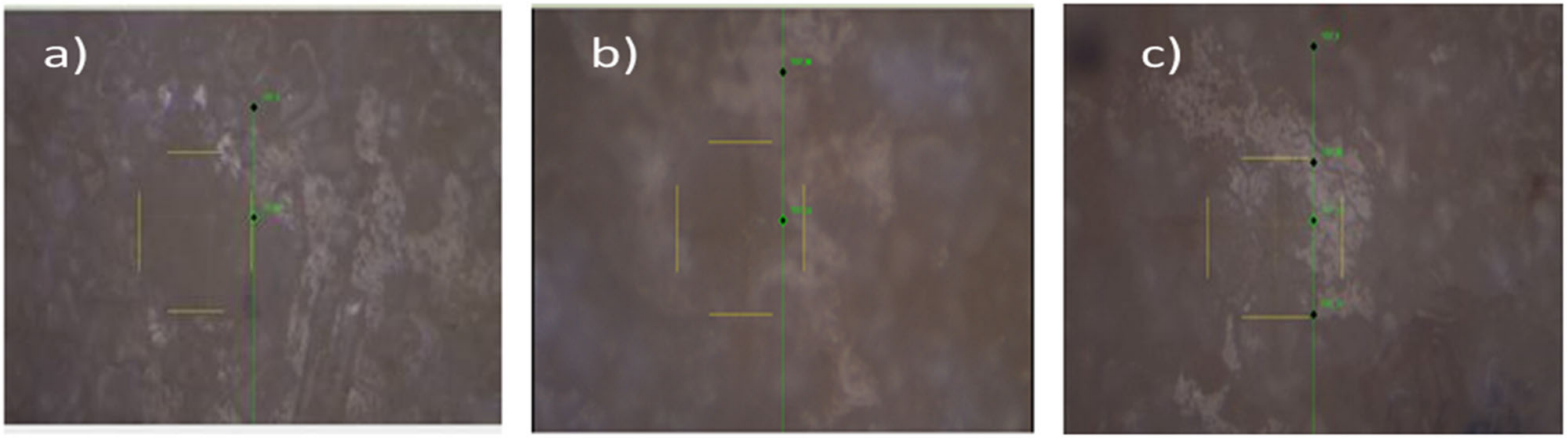
Optical microscopic images of (**a**) 3 M%, (**b)** 4 M% and (**c)** 5 M% YSZ coatings

**Fig. 11:**
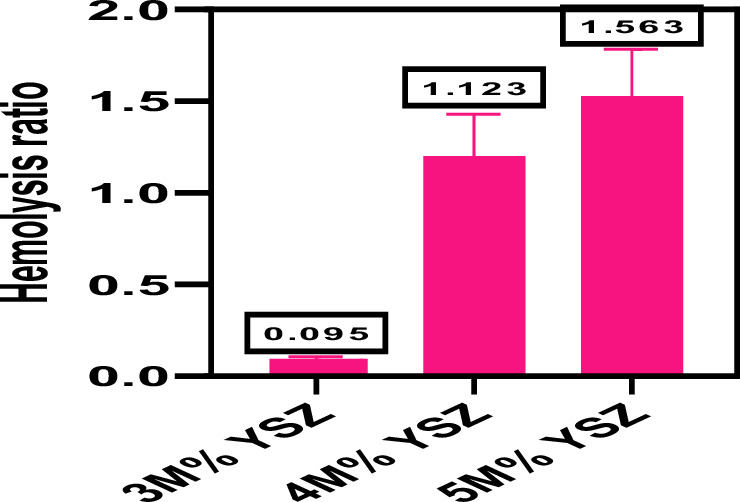
The hemolysis percentage of 3 M%, 4 M% and 5 M% YSZ composites

The mechanical characteristics of plasma-sprayed YSZ coatings with varying molar concentrations of Y_2_O_3_ (3, 4, and 5 M%) were assessed using the Vickers microhardness test (Fig. 9, 10). Indentations were performed at a load of 0.5 kgf with a dwell time of 15 seconds, and three measurements were averaged per sample. All coatings demonstrated strong adhesion to the metallic substrate, with no signs of delamination, indicating interfacial bonding due to the effective anchoring of molten particles during the plasma spraying process [39]. The Vickers hardness (VHN) values increased with the Y_2_O_3_ content, measured at 1.53 ± 1.30 GPa for 3 M%, 2.33 ± 0.30 GPa for 4 M%, and 4.13 ± 0.14 GPa for 5 M% YSZ coatings. This progressive improvement in mechanical strength is attributed to the stabilization of the non-transformable tetragonal and cubic zirconia phases by Y_2_O_3_ doping. Cheng Qian et al. emphasized the necessity of high-temperature sintering (above 1200 °C) to enhance the densification and mechanical performance of YSZ coatings [40]. In the present study, although the powders were sintered at 1200 °C prior to deposition, complete transformation from the monoclinic to tetragonal phase was limited in the 3 M% and 4 M% coatings. However, the 5 M% YSZ coating exhibited a dominant presence of the stable tetragonal and cubic phases, as corroborated by phase analysis. The mechanical superiority of the 5 M% YSZ coating is also reinforced by its microstructural uniformity and reduced porosity, as observed in SEM images. Increased Y_2_O_3_ doping leads to the suppression of oxygen vacancies and the inhibition of crack propagation by stabilizing the high-temperature phases, ultimately enhancing toughness [41, 42]. Moreover, the fine grain structure and absence of spallation or microcracks contribute to its improved hardness and mechanical integrity [43]. Zhelun et al. reported that YSZ coatings with higher Y_2_O_3_ concentrations showed better mechanical properties due to enhanced intergranular cohesion and phase stability [44]. Thus, among the tested compositions, the 5 M% YSZ coating demonstrates the most promising mechanical behavior, with high hardness, phase stability, and minimal surface defects making it highly suitable for orthopedic and dental implant applications where long-term mechanical reliability is critical Figs. 9–11.

### Hemocompatibility

The hemocompatibility of yttria-stabilized zirconia (YSZ) composites with varying Y_2_O_3_ concentrations (3 M%, 4 M%, and 5 M%) was evaluated using the ASTM F756-00 [45] standard test for hemolysis. This assay assesses the degree of red blood cell (RBC) lysis upon contact with material surfaces. According to ASTM F756-00, the hemolytic ratio is classified into three categories: >5% Hemolytic, 2–5% slightly hemolytic, <2% non-hemolytic. The hemolytic ratio was calculated by measuring the absorbance of released hemoglobin at 540 nm using a UV–Vis spectrophotometer. The results showed that the hemolytic ratios for the YSZ coatings were: 3 M% YSZ: 0.095%, 4 M% YSZ: 1.123%, 5 M% YSZ: 1.563%. All values were below the 2% threshold, confirming that all three compositions are non-hemolytic and exhibit excellent hemocompatibility (Fig. 11). No visible hemoglobin traces were observed in any of the samples, and no morphological evidence of RBC lysis or damage was detected. Although a slight increase in the hemolytic ratio was noted with increasing Y_2_O_3_ content, it remained within the safe, non-hemolytic range. This mild increase may be associated with subtle changes in surface properties such as hydrophilicity or roughness, but no ion leaching was observed, indicating the chemical stability of YSZ in physiological environments. The absence of hemolysis confirms that yttrium dopant ions (Y^3+^) do not interact adversely with the RBC membrane, supporting the biocompatibility and blood compatibility of YSZ composites. These findings reinforce previous reports that YSZ ceramics are bioinert, hemocompatible, and chemically stable, making them highly suitable for orthopedic and dental implants that may come into contact with blood [46, 47].

### Antibacterial evaluation

**Fig. 12:**
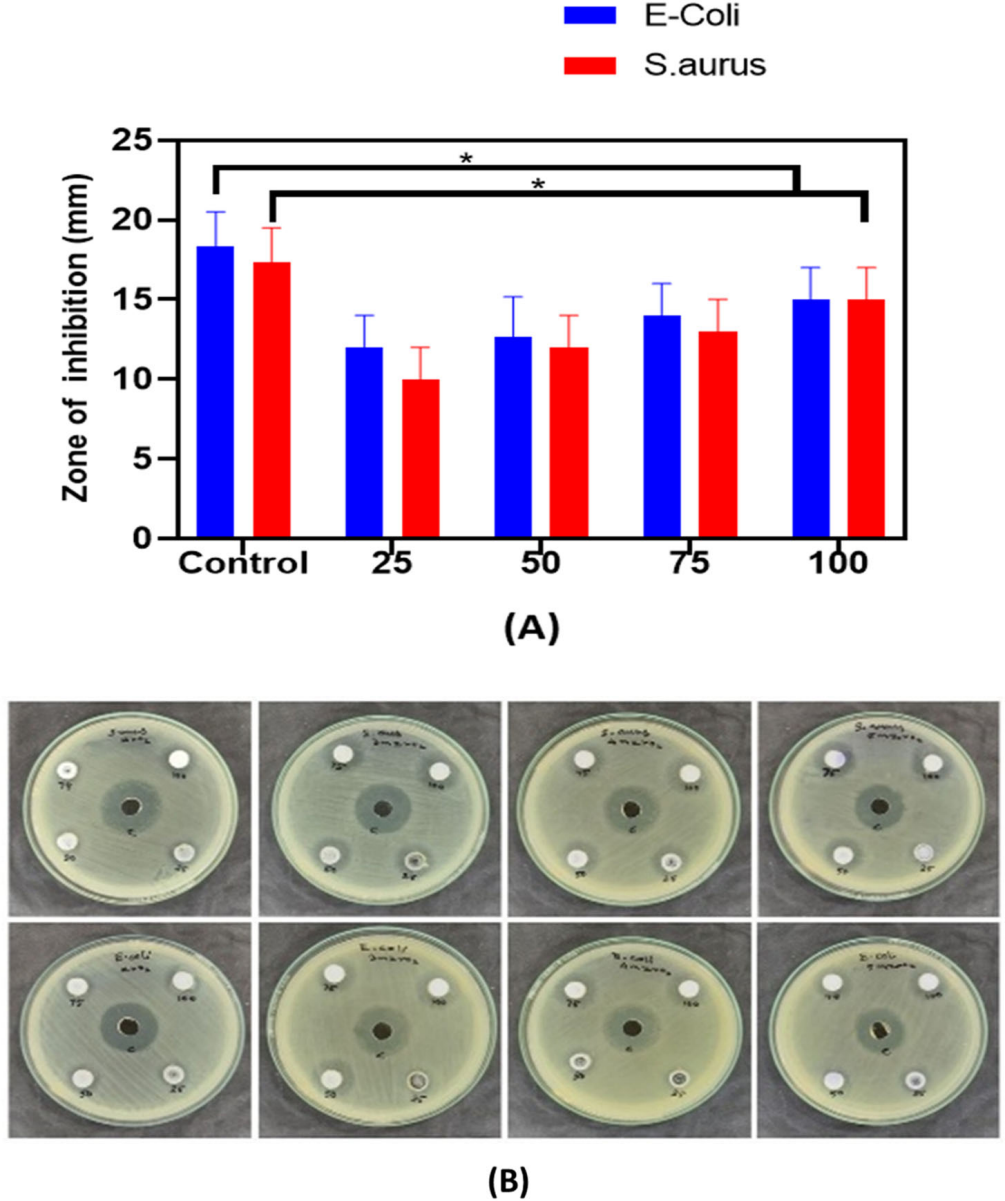
**A** Zone of inhibition of 5 M% YSZ composite. The (*) represents that the difference is statistically significant at *p* < 0.05 (data represent the mean and ± standard deviation of 5 M% YSZ composite), **B** Antibacterial assessment by disc diffusion method of *S. aureus and E-coli* (ZrO_2_, 3 M%YSZ, 4 M% YSZ, 5 M% YSZ) composites

The antibacterial efficacy of pure zirconia (ZrO_2_) and yttria-stabilized zirconia (YSZ) composites which is used for coating was systematically investigated using the agar well diffusion method against *Escherichia coli* (Gram-negative) and *Staphylococcus aureus* (Gram-positive), representing typical bacterial threats in implant-associated infections. As shown in Fig. 12A show the antibacterial activity of pure zirconia and YSZ containing 3 M%, 4 M% and 5 M% showed significantly lower values compared to the positive control group (data represent the mean ± standard deviation of Pure zirconia, 3 M% YSZ, 4 M% YSZ, 5 M% YSZ composites (*p* < 0.05). Pure ZrO_2_ demonstrated minimal antibacterial activity, particularly against *S. aureus*, which is consistent with previous studies indicating that undoped zirconia lacks intrinsic antimicrobial properties due to its chemical inertness and low ion release capability [48, 49]. However, doping with yttria significantly enhanced the antibacterial performance. Figure 12B zone of inhibition against *E. coli* increased from 10 mm (25 µl) to 15 mm (100 µl) for the 5 M% YSZ, compared to only 12 mm for pure ZrO_2_ at the same concentration. A similar behavior was observed for *S. aureus*, where the inhibition zone reached 15 mm at 100 µl for 5 M% YSZ. These results correlate with the work of Gopinath et al. [50] and Priyadharshini et al., [51], who reported enhanced bacterial inhibition with increasing Y_2_O_3_ concentration in zirconia due to improved ion release and surface charge properties. The order of antibacterial activity follows: *E. coli*: 5 M% YSZ > 4 M% YSZ > 3 M% YSZ > Pure ZrO_2_, *S. aureus*: 5 M% YSZ > 4 M% YSZ > 3 M% YSZ > Pure ZrO_2_. The enhanced antibacterial action of 5 M% YSZ can be attributed to a synergistic mechanism involving both ion release and reactive oxygen species (ROS) generation. Upon contact with bacterial cells, Y^3+^ ions are released from the surface and interact with the negatively charged cell membranes, leading to membrane destabilization and cytoplasmic leakage [52]. Furthermore, Y^3+^ ions may bind to the bacterial DNA, interfering with replication and metabolic processes, ultimately resulting in cell death. In addition to ion toxicity, ROS generation especially superoxide anions (O_2_^−^) and hydroxyl radicals (•OH) plays a pivotal role in bactericidal activity. [53, 54].

Furthermore, studies have shown that YSZ’s surface roughness and hydrophilicity can influence bacterial adhesion. The 5 M% YSZ exhibited lower contact angles and higher surface energy, making it more hydrophilic conditions that can enhance antibacterial properties by facilitating ROS interaction and ion mobility [55, 56]. Although rougher surfaces can sometimes support bacterial colonization, in this case, the optimized microstructure of 5 M% YSZ with fewer voids and strong grain cohesion reduces the available surface area for bacterial adhesion. Another contributing factor is slow degradation kinetics in DMSO and other physiological solvents, allowing for sustained release of Y^3+^ ions over time. Dapunt et al. [57] demonstrated that YSZ coatings maintain antimicrobial activity over prolonged periods due to controlled yttrium ion leaching, without compromising structural integrity. In terms of clinical relevance, implant associated infections are a significant cause of implant failure. Materials with inherent or engineered antibacterial properties are being sought after to reduce biofilm formation and microbial colonization [58]. The present results clearly establish 5 M % YSZ as a bio ceramic with dual functionality mechanical strength and antibacterial efficacy making it a strong candidate for orthopedic and dental implant coatings.

### Biocompatibility assay

**Fig. 13:**
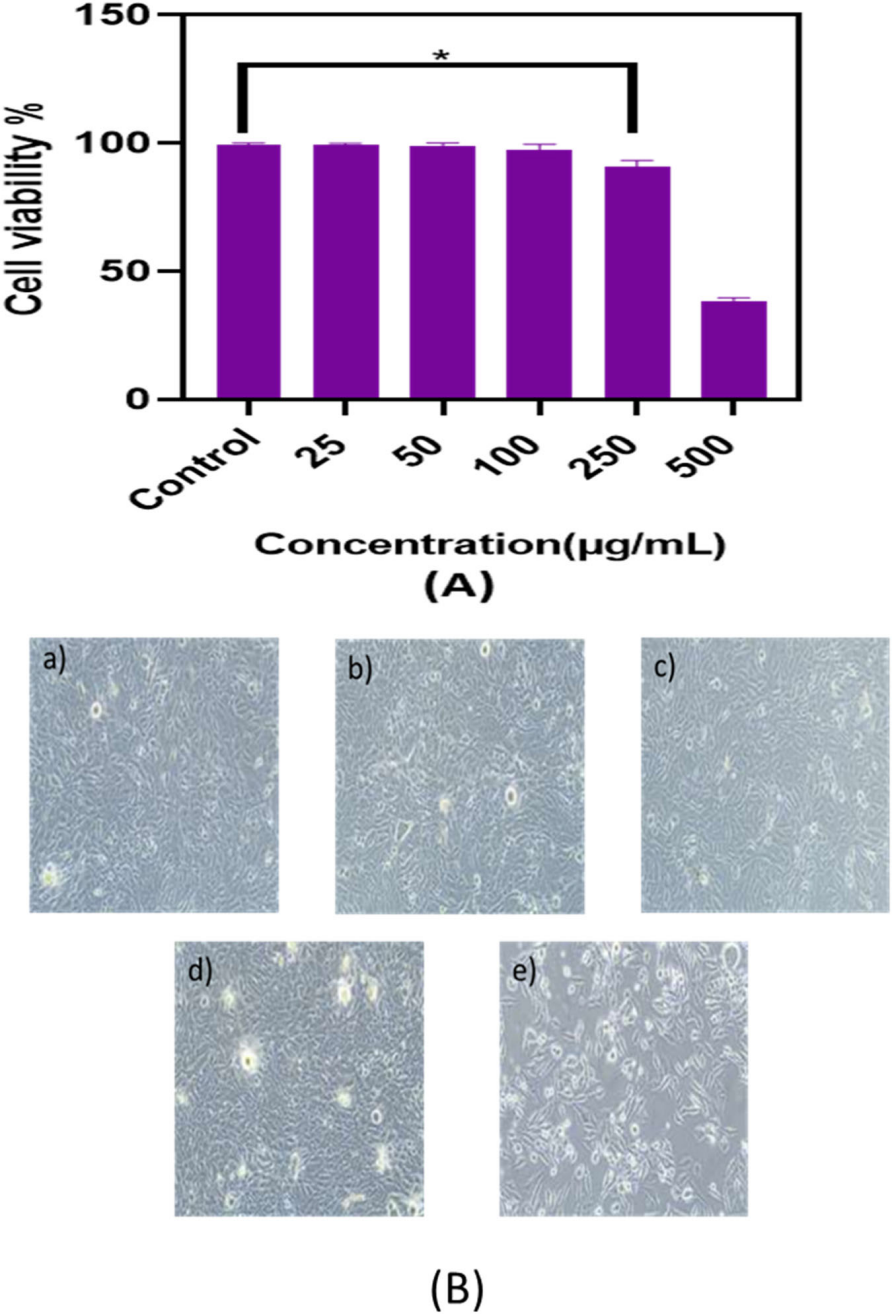
**A** Percentage of cell viability. The (*) represents that the difference is statistically significant at *p* < 0.01 (data represent the mean and ± standard deviation of 5 M% YSZ composite). **B** In vitro cell viability of 5 M% YSZ at various concentration **a** 25 µg/ml, **b** 50 µg/ml, **c** 100 µg/ml, **d** 250 µg/ml, **e** 500 µg/ml using MG-63 cell line

The cytocompatibility of yttria-stabilized zirconia (YSZ) composites with varying Y_2_O_3_ content (3 M %, 4 M%, and 5 M%) was assessed using the MTT assay on MG-63 osteoblast-like cells. Cells were exposed to YSZ suspensions at concentrations ranging from 25 to 500 µg/ml for 24 h. MTT results, in Fig. 13A, demonstrated that cell viability of pure zirconia and 3 M%, 4 M%, and 5 M% YSZ, all of which exhibited significantly lower values compared to the positive control group. The data represent the mean ± standard deviation for pure zirconia and YSZ composites (3 M%, 4 M%, and 5 M%) up to a concentration of 250 µg/ml (*p* < 0.01). YSZ compositions supported good cell viability at lower concentrations (25–250 µg/ml), indicating non-toxic behavior and cellular compatibility across the tested range. The cell viability remained above 85% at these lower concentrations, confirming biocompatibility according to ISO 10993-5 standards. However, at 500 µg/ml, a slight reduction in cell viability was observed, with values declining to ~70–75%. This cytotoxic response at higher concentrations is attributed to localized Y^3+^ ion release, which may disrupt intracellular metabolic activity and compromise membrane function [59–61].

Phase contrast microscopy images Fig. 13B, revealed healthy cell attachment, spreading, and proliferation on YSZ surfaces at 25–250 µg/ml. Live cells exhibited elongated, spindle-like morphology typical of healthy osteoblasts, whereas a minor population of rounded, non-adherent cells was visible at 500 µg/ml, supporting the observed cytotoxicity at higher doses. Quantitative data presented in Table 5 reinforce the conclusion that 3 M%, 4 M%, and 5 M% YSZ are cytocompatible, especially at lower doses. These results are consistent with existing literature indicating that yttria-stabilized zirconia enhances osteoblast activity and adhesion at low concentrations [55, 62], excessive dopant ion release may impact mitochondrial activity and protein expression at higher doses [63, 64], surface roughness and grain structure also play a role in modulating MG-63 cell response [56, 65]. Thus, YSZ composites containing up to 5 M% Y_2_O_3_ demonstrate excellent cytocompatibility and are suitable for orthopedic and dental implant applications.

**Table 5:** Absorbance and % cytotoxicity of YSZ powder

Tested concentration(µg/ml)	OD at 570 nm (triplicate value)			% of cell viability		
**Control**	0.327	0.334	0.332	99.98	99.98	99.99
25	0.325	0.330	0.330	99.09	99.86	99.95
50	0.324	0.332	0.320	97.89	99.70	99.80
100	0.319	0.325	0.331	96.37	100.00	96.68
250	0.300	0.310	0.296	90.63	93.66	89.43
500	0.129	0.121	0.130	38.97	36.56	39.27

## Conclusion

The following conclusion are examined based on the results obtained from the studies.

We have successfully fabricated YSZ at various concentration by the co- precipitation technique, and the resultant powder was used for coating on Ti-6Al-4V alloy by Plasma spray technique.

1. The non-transformable phases such as tetragonal and cubic phases were achieved at the concentration of 5 M% which is considered as preferable phase.
2. The morphology and thickness of the coating was optimised by varying the process parameters of plasma spray technique. The optimised 5 M% was found to be uniform and crack free with the thickness of 108.8 µm.
3. The hardness of 4.13 ± 0.14Gpa and surface roughness of 14.13 ± 1.5 of the coating was obtained for the optimised coating.
4. It is noteworthy that the antibacterial activity against gram-positive and gram-negative bacteria showed excellent antibacterial property.
5. The cell viability and proliferation efficiency using MG-63 osteoblast cells on 3–5 M% of YSZ composites were found to be non-toxic up to 250 µg/ml whereas at higher concentration, the viability of cells is slightly decreased.

At present 3 M% of YSZ ceramics are the most often used ceramic material in the biomedical industry for orthopedic and dental applications. From this work, 5 M% of YSZ concentration was optimised as crack free with require adhesion strength using plasma spray technique, for the effective use in biomedical application.
